# Safety and efficacy of laparoscopic radical gastrectomy after neoadjuvant chemotherapy plus immunotherapy: a retrospective cohort study

**DOI:** 10.3389/fimmu.2025.1672547

**Published:** 2025-09-04

**Authors:** Junjie Xiong, Yu Zou, Yunlong Zhang, Fan He, Chenglin Tang, Kemei Zhong, Kun Qian

**Affiliations:** Department of Gastrointestinal Surgery, The First Affiliated Hospital of Chongqing Medical University, Chongqing, China

**Keywords:** neoadjuvant chemotherapy, immunotherapy, pathologic complete response, locally advanced gastric cancer, laparoscopic radical gastrectomy

## Abstract

**Background:**

Gastric cancer (GC) is a highly prevalent type of malignant tumor worldwide. Patients with locally advanced gastric cancer (LAGC) frequently have a poor prognosis due to the inability to achieve R0 resection. Neoadjuvant chemotherapy (NAC) can enhance survival rates, although its effectiveness is limited. Immune checkpoint inhibitors (ICIs) have demonstrated potential in treating advanced gastric cancer, but their efficacy in neoadjuvant therapy (NAT) for LAGC remains unclear. The aim of this study was to evaluate the safety, pathological response and survival outcome of neoadjuvant chemotherapy plus immunotherapy (NACI) versus NAC alone after laparoscopic gastrectomy for GC.

**Methods:**

A retrospective analysis of 375 patients with LAGC who received neoadjuvant therapy from 2015 to 2022 was performed. Patients were divided into NACI group (168 patients) and NAC group (207 patients) according to NAT regimen.

**Results:**

The rate of pathologic complete response (pCR, 20.2% vs. 12.6%, P=0.04) and the rate of major pathological response (MPR, 31.0% vs. 18.8%, P=0.007) in the NACI group are significantly higher than those in the NAC group, and the NACI group also had a higher rate of R0 resection (91.3% vs. 84.1%, P=0.028). The NACI group experienced a more significant decline in ypT0 (22.0% vs. 13.0%, P=0.022) and ypN0 (67.3% vs. 53.6%, P=007), but there was no difference in disease-free survival (DFS) and overall survival (OS) at 3 years between the two groups (P>0.05).

**Conclusions:**

NACI significantly improved pCR rates and R0 resection rates in patients with LAGC without increasing perioperative risk, but did not translate into short-term survival benefits.

## 1 Introduction

Recent epidemiological studies indicate that gastric cancer ranks as the fifth most common malignancy globally, both in terms of incidence and mortality rates (1). Owing to the insidious onset and nonspecific early symptoms of gastric cancer (GC), the majority of patients present with advanced-stage disease at diagnosis, frequently precluding curative R0 resection. This clinical challenge contributes to elevated postoperative recurrence rates and diminished OS. Although surgical resection remains the cornerstone of GC therapy, its standalone efficacy is often suboptimal, underscoring the need for multimodal treatment strategies (2–4). Consequently, conversion therapy has emerged as a pivotal research focus for LAGC, aiming to enable radical resection and improve outcomes in initially unresectable cases.

The landmark 2006 MAGIC trial (5) first established that NAC significantly improves OS and DFS compared to surgery alone in gastric cancer patients. These findings were subsequently validated in large-scale Phase III trials, including Ychou et al.’s 2011 study (6) and Al-Batran et al.’s Phase II/III trial (7) in 2019, confirming the survival benefits of NAC in multimodal treatment approaches. Consequently, the multimodal strategy combining NAC with surgical resection has gained widespread clinical adoption for LAGC. This integrated approach demonstrates three key advantages (1): significant tumor downstaging (2), improved R0 resection rates, and (3) enhanced long-term survival outcomes, all achieved without increasing postoperative morbidity or mortality. These evidence-based benefits have established NAC followed by surgery as a cornerstone therapeutic paradigm for LAGC management (8–10).

ICIs have revolutionized oncology practice, emerging as first-line therapeutics for multiple malignancies. Their remarkable efficacy has facilitated the integration of immunotherapy into comprehensive cancer treatment paradigms, where they now synergize with established modalities including surgical resection, chemotherapy, radiotherapy, and molecularly targeted agents (11). Programmed cell death protein 1 (PD-1) represents one of the most extensively characterized immune checkpoint receptors, playing a pivotal role in the precise modulation of T cell activation and the maintenance of immune homeostasis. As a key regulator of peripheral tolerance, PD-1 mediates intrinsic immunosuppressive signals that normally prevent excessive immune activation. However, tumors co-opt this physiological regulatory mechanism through sustained PD-1 pathway activation, creating an immunosuppressive tumor microenvironment that promotes T cell exhaustion and facilitates malignant immune evasion (12, 13). The clinical success of PD-1 monoclonal antibodies has revolutionized cancer immunotherapy, establishing a new paradigm in oncology. Accumulating evidence from multiple randomized trials demonstrates that PD-1 inhibitors, particularly in combination with chemotherapy, have emerged as a cornerstone treatment for unresectable advanced or recurrent gastric/gastroesophageal junction cancers, significantly improving DFS. Nevertheless, their impact on OS continues to be investigated, with current data showing heterogeneous results across clinical studies (14–18).

While PD-1 inhibitors have established favorable efficacy and safety profiles in unresectable/recurrent gastric cancer, their application in neoadjuvant settings for LAGC remains investigational. The therapeutic potential and safety concerns of combining PD-1 blockade with neoadjuvant chemotherapy in LAGC require systematic evaluation. This study investigates this novel combinatorial approach, aiming to elucidate its clinical benefits and establish a new therapeutic paradigm for LAGC management.

## 2 Method

### 2.1 Study population and design

This retrospective cohort study evaluated consecutive LAGC patients undergoing NAT followed by curative-intent surgery at The First Affiliated Hospital of Chongqing Medical University (January 2015-December 2022). Since the first patient in our hospital underwent NACI surgery in July 2021, patients in the NACI group were enrolled from July 2021 to December 2022. Inclusion required (1): histologically confirmed LAGC (cT1-2N+M0 and cT3-4N×M0) (2); no prior/concurrent malignancies (3); imaging and clinical assessments confirming no distant metastasis or direct invasion of adjacent organs; and (4) R0 resection completion. Key exclusions comprised (1): prior gastric resection (2); acute cardiovascular events (≤3 months) (3); emergency surgery (4); incomplete adjuvant chemotherapy (5); undocumented NAT regimens; or (6) inadequate follow-up. After rigorous screening, 559 patients comprised the final analytical cohort.

This retrospective study was conducted in accordance with the ethical standards of the Declaration of Helsinki and received formal approval from the Institutional Review Board of The First Affiliated Hospital of Chongqing Medical University (Approval No. 2025-019-01). The ethics committee waived the requirement for informed consent due to the retrospective nature of the study and use of anonymized clinical data.

### 2.2 Neoadjuvant therapy and surgical intervention

Based on the differences in NAT, the primary classification divides it into two groups: the Oxaliplatin plus S-1 group (NAC) and the PD-1 inhibitors combined with SOX group (NACI). The NAC treatment cycle is as follows: on Day 1, intravenous infusion of Oxaliplatin at 130 mg/m²; from Day 1 to Day 14, oral administration of S-1, with dosages adjusted based on body surface area—120 mg/day for BSA ≥ 1.5 m², 100 mg/day for BSA between 1.25 and 1.5 m², and 80 mg/day for BSA < 1.25 m², administered twice daily. Each cycle spans 21 days. The NACI treatment cycle is as follows: on Day 1, intravenous infusion of oxaliplatin at 130 mg/m² and a PD-1 inhibitor at 200 mg (Sintilimab, Camrelizumab and Tislelizumab) or 360mg(Nivolumab); from Day 1 to Day 14, oral administration of S-1, with dosages based on body surface area—120 mg/day for BSA ≥1.5 m², 100 mg/day for BSA between 1.25 and 1.5 m², and 80 mg/day for BSA <1.25 m², taken twice daily. Each cycle lasts 21 days. Patients should undergo curative gastrectomy within 3 to 6 weeks following completion of neoadjuvant therapy. The surgical approach primarily involves laparoscopic procedures. Depending on tumor size, location, and regional lymph node involvement, either total or partial gastrectomy (distal or proximal) should be performed. A D2 or D2+ lymphadenectomy is recommended, and organ resection may be incorporated as necessary to achieve an R0 resection (19).

### 2.3 Data

A retrospective extraction of clinical, pathological, and follow-up data from the gastric cancer database maintained by the First Affiliated Hospital of Chongqing Medical University. The dataset includes demographic parameters such as gender, age at diagnosis, body mass index (BMI) and Eastern Cooperative Oncology Group (ECOG) performance status. Additionally, tumor-related and perioperative variables were collected, including tumor location, preoperative imaging-based TNM staging, pathological ypTNM staging, tumor regression grade (TRG), resection margin status (R0/R1), presence of vascular and nerve invasion, operative duration, intraoperative blood loss, postoperative complications, and length of hospital stay.

### 2.4 End points and assessments

The primary endpoint of the study is the pCR rate. Secondary endpoints include MPR rate, DFS, OS, ypTNM staging, total number of lymph nodes examined, number of positive lymph nodes, surgical complications, and adverse reactions to NAT.

Patients undergo clinical response assessments every 21 days. Localized CT scans are performed before and after NAT, as well as postoperatively. Tumor response to NAT is evaluated by comparing pre- and post-therapy CT scans in accordance with RECIST 1.1 criteria. Pathological response of gastric resection specimens was locally assessed by pathologists from participating hospitals. DFS is defined as the interval from the date of surgery to the first recorded occurrence of tumor recurrence, metastasis, or death. OS is defined as the time from the date of surgery to death. R0 resection is characterized by complete tumor removal with no gross or microscopic residual disease, whereas R1 resection indicates macroscopically complete tumor excision with microscopic involvement of the resection margins. TRG is assessed according to the Becker criteria: TRG 1a (no residual tumor cells), TRG 1b (<10% residual tumor cells), TRG 2 (10-50% residual tumor cells), and TRG 3 (>50% residual tumor cells). Achieving TRG 1a is defined as pCR. MPR is defined as having less than 10% residual tumor cells, with TRG 1a/1b classified as MPR (20–22). Post-chemotherapy complications are defined as adverse events occurring during the course of chemotherapy and are documented according to the Common Terminology Criteria for Adverse Events (CTCAE) version 5.0 (23). Postoperative complications are characterized as adverse events arising during the postoperative hospitalization period and are graded using the Clavien-Dindo classification system (23–25). Baseline patient data, perioperative information, and pathological data during hospitalization were extracted from the electronic medical record system. The number of chemotherapy cycles was determined by referencing hospital medical records or through telephone follow-up. DFS and OS were obtained via review of previous inpatient and outpatient records, supplemented by telephone follow-up. The follow-up period extended from the date of surgery to April 2025.

### 2.5 Statistical analysis

All data were analyzed using IBM SPSS Statistics 27.0, with survival curves generated via GraphPad Prism 10.5. Continuous variables with a normal distribution were analyzed using independent samples t-tests and expressed as means ± standard deviations, while non-normally distributed continuous variables were analyzed with the Mann-Whitney U test and presented as medians with interquartile ranges. Categorical variables were analyzed using χ² tests, Fisher’s exact test, or Mann-Whitney U tests, with results expressed as frequencies and percentages. Logistic regression analysis was employed to evaluate factors influencing TRG (1a+1b). Survival analysis was conducted using the log-rank test, and Kaplan-Meier curves were plotted accordingly. A p-value of less than 0.05 was considered statistically significant, and Cox regression analysis was performed to identify factors associated with survival outcomes.

## 3 Results

### 3.1 Patient disposition and baseline characteristics

**Figure 1:**
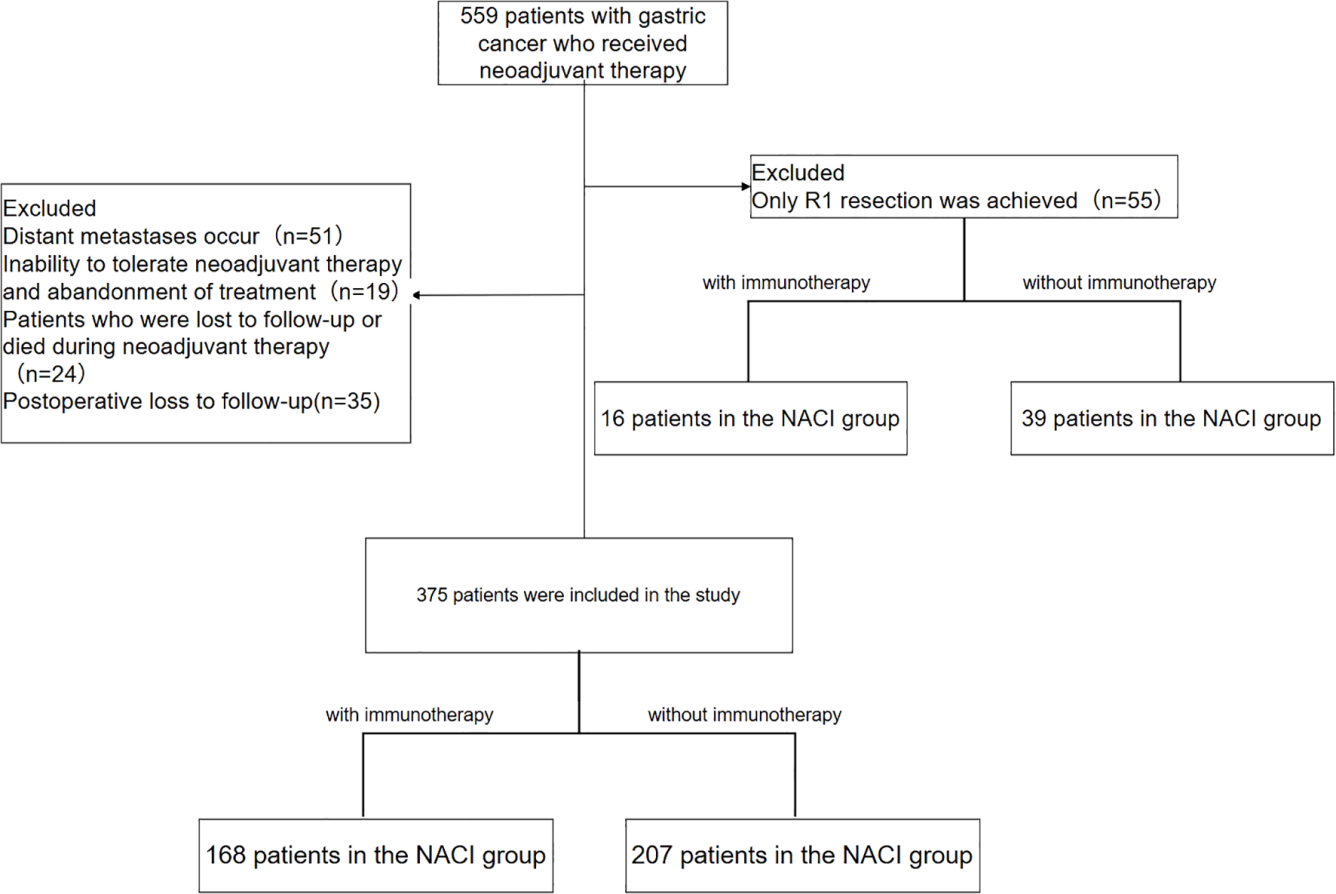
Patient inclusion flowchart.

In this retrospective study, we evaluated 559 gastric cancer patients who underwent neoadjuvant therapy between January 2015 and December 2022. Since the first patient in our hospital underwent NACI surgery in July 2021, patients in the NACI group were enrolled from July 2021 to December 2022. After exclusion criteria were applied—including distant metastasis precluding curative surgery (n = 51), treatment discontinuation due to therapy intolerance (n = 19), loss to follow-up or death during neoadjuvant treatment (n = 24), R1 resection (n = 55; NAC group: 39, NACI group: 16), and postoperative loss to follow-up (n = 35)—a final cohort of 375 patients was analyzed. This cohort consisted of 207 patients in the NAC group and 168 in the NACI group (**Figure 1**).

No significant differences were observed between the two groups in baseline demographic or clinical characteristics, including sex, age, BMI, comorbidities, tumor location, pretreatment TNM stage, ECOG performance status, or the number of neoadjuvant therapy cycles (all P > 0.05) (**Table 1**).

**Table 1:** Baseline demographic and clinical characteristics of patients before surgery.

Baseline variable	NAC group	NACI group	χ^2^/Z	P value
	(n=207) %	(n=168) %		
Gender			χ²=0.174	0.677
Male	140(67.6%)	117(56.5%)		
Female	67(32.3%)	51(43.5%)		
Age			Z=-0.61	0.951
Median (IQR), year	61(53,69)	60(52.25,69.00)		
BMI			Z=-0.624	0.533
Median (IQR), kg/m2	22.07(19.96,24.98)	22.41(19.84,25.24)		
Comorbidity	39(18.8%))	35(20.8%)	χ²=0.232	0.630
Hypertension	20(9.6%)	18(10.7%)	χ²=0.113	0.737
Diabetes	20(9.6%)	17(10.1%)	χ²=0.022	0.883
Coronary disease	3(1.4%)	6(3.5%)	χ²=1.783	0.182
COPD	2(0.9%)	0(0.0%)	Fisher	0.201
Hepatopathy	2(0.9%)	2(1.1%)	χ²=0.000	1
Tumor location			χ²=2.804	0.246
Upper	44(21.2%)	39(23.2%)		
Middle	63(30.5%)	62(36.9%)		
Lower	100(48.3%)	67(39.9%)		
cT stage			Z=-0.092	0.926
T1	0(0.0%)	1(0.6%)		
T2	1(0.5%)	7(4.2%)		
T3	54(26.1%)	34(20.2%)		
T4	152(73.4%)	126(75.0%)		
cN stage			χ²=0.060	0.807
N0	39(18.8%)	30(17.8%)		
N1-3	168(81.2%)	138(82.2%)		
ECOG score			Z=-1.175	0.519
0	183(88.4%)	152(90.5%)		
1	21(10.2%)	14(8.3%)		
2	3(1.4%)	2(1.2%)		
Neoadjuvant cycles			χ²=1.667	0.197
≤3	180(86.9%)	138(82.2%)		
>3	27(13.1%)	30(17.8%)		

BMI, Body mass index; COPD, chronic obstructive pulmonary disease; NAC, neoadjuvant chemotherapy; NACI, Neoadjuvant chemotherapy plus immunotherapy.

Negative Z for Age and BMI indicates “NACI group” < “NAC group”.

### 3.2 Surgical factors and postoperative complications

Surgical safety profiles were comparable between the two groups, with no significant differences in resection method, gastrointestinal reconstruction technique, operative time, intraoperative blood loss, timing of drain removal, gastric tube placement/removal time, or postoperative complication rates (all P > 0.05) (**Table 2**).

**Table 2:** Surgical outcomes and postoperative complications.

Baseline variable	NAC group	NACI group	χ²/Z	P value
	(n=207) %	(n=168) %		
Extent of resection			χ²=1.286	0.526
Distal gastrectomy	82(39.6%)	57(33.9%)		
Total gastrectomy	115(55.6%)	102(60.7%)		
Proximal gastrectomy	10(4.8%)	9(5.4%)		
Reconstruction method			χ²=13.125	0.11
Billroth-1	10(4.8%)	3(1.8%)		
Billroth-2	64(30.9%)	40(23.8%)		
Roux-en-Y	106(51.2%)	105(62.5%)		
Esophago-Gastric Anastomosis	1(0.5%)	6(3.6%)		
splt Roux-en-Y	26(1265%)	14(8.3%)		
Duration of operation	225(185,269)	225(171.25,290)	Z=-0.457	0.648
Median (IQR), min				
Estimated blood loss	100 (50,200)	100 (50,165)	Z=-0.477	0.634
Median (IQR), ml				
Postoperative hospital stay	12(9,16)	12(10,14.75)	Z=-1.679	0.093
Median (IQR), day				
Duration of abdominal drain indwelling	8(7,10)	8(7,11)	Z=-0.514	0.607
Median (IQR), day				
Intraoperative placement of a gastric tube			χ²=1.912	0.166
Yes	187(90.4%)	144(85.7%)		
No	20(9.6%)	24(14.3%)		
Duration of indwelling gastric tube	5(4,6)	5(4,6)	Z=-1.802	0.072
Median (IQR), day				
Complications			χ²=0.034	0.853
Yes	36(17.4%)	28(16.7%)		
Anastomotic leakage	9(4.3%)	10(5.9%)	χ²=0.496	0.481
Intra-abdominal infection	16(7.7%)	7(4.1%)	χ²=2.045	0.153
Lung infection	7(3.3%)	4(2.3%)	χ²=0.069	0.792
Lymphatic leakage	0(0.0%)	1(0.6%)	Fisher	0.448
Pancreatic leakage	0(0.0%)	1(0.6%)	Fisher	0.448
hemorrhage	5(2.4%)	9(5.3%)	χ²=2.233	0.135
Incision infection	3(1.4%)	0(0%)	χ²=0.968	0.325
Anastomotic narrow	0(0%)	1(0.6%)		0.448
Clavien-Dindo grading			Z=-0.205	0.837
0	171(82.6%)	140(83.3%)		
1	3(1.4%)	2(1.2%)		
2	29(14.0%)	24(14.3%)		
3	3(1.4%)	2(1.2%)		
4	1(0.5%)	0(0%)		

NAC, neoadjuvant chemotherapy; NACI, Neoadjuvant chemotherapy plus immunotherapy.

The Z of Duration of operation, Estimated blood loss, Postoperative hospital stay, Duration of abdominal drain indwelling and Duration of indwelling gastric tube is negative for “NACI group” < “NAC group”.

### 3.3 Changes in tumor markers and adverse treatment reactions

Comparative analysis of tumor markers and hematological parameters before and after neoadjuvant therapy demonstrated significant reductions in serum CEA and CA19-9 levels in both the NACI and NAC groups (P < 0.05), while AFP levels didn’t change significantly (P > 0.05). Hematological profiling revealed treatment-induced alterations in white blood cell count, neutrophil count, hemoglobin levels, platelet count, ALT, and AST (all P < 0.05). Notably, lymphocyte counts were unaffected by either treatment regimen (P > 0.05) (**Table 3**).

**Table 3:** Changes in tumor markers and hematological parameters before and after neoadjuvant therapy.

	NAC		χ²/Z	P value	NACI		χ²/Z	P value
	First diagnosed	Preoperative			First diagnosed	Preoperative		
CEA			Z=-2.198	0.005			Z=-2.664	0.008
Median (IQR), ng/mL	2.6(1.7,6.1)	2.8(1.9,4.1)			2.65(1.6,6.1)	2.3(1.725,4.0)		
CA19-9			Z=-2.648	0.008			Z=-2.910	0.004
Median (IQR), U/mL	9.0(5.3,19.99)	9.8(5.3,16.0)			12.85(6.300,33.625)	9.9(5.15,16.8)		
AFP			Z=-1.360	0.174			Z=-1.116	0.264
Median (IQR), ng/mL	3.0(2.2,4.0)	3.0(2.2,4.23)			2.6(1.925,3.975)	2.9(2.025,4.25)		
Leukocyte			Z=-5.910	<0.001			Z=-5.352	<0.001
Median (IQR), ×10^9^/L	5.51(4.51,6.51)	4.86(4.00,5.56)			5.42(4.22,6.53)	4.45(3.49,5.55)		
Neutrophils			Z=-6.643	<0.001			Z=-7.057	<0.001
Median (IQR), ×10^9^/L	3.48(2.67,4.53)	2.79(1.91,3.58)			3.30(2.48,4.34)	2.35,(1.85,3.178)		
Lymphocyte			Z=-0.740	0.459			Z=-0.395	0.693
Median (IQR), ×10^9^/L	1.3(1.05,1.63)	1.35(1.35,1.66)			1.33(1.02,1.80)	1.34(0.99,1.74)		
Hemoglobin			Z=-2.686	0.007			Z=-4.7	<0.001
Median (IQR), g/L	116(95,131)	111(98,124)			115(96.5,133.0)	108(95.5,120.0)		
Platelets			Z=-9.524	<0.001			Z=-8.131	<0.001
Median (IQR), ×10^9^/L	231(185,291)	188(132,236)			218.5(175.25,292.75)	168(130.75,212.75)		
ALT			Z=-3.168	0.002			Z=-5.920	<0.001
Median (IQR), U/L	19(12,26)	22(16,29)			15(11,21)	20(14,31)		
AST			Z=-5.333	<0.001			Z=-7.216	<0.001
Median (IQR), U/L	19(16,25)	24(20,29)			17(14,24)	23(19,33.75)		

CEA, Carcinoembryonic antigen; CA199, Carbohydrate antigen199; AFP, Alpha-fetoprotein; ALT:alanine aminotransferase; AST, Aspartate aminotransferase; Z is a negative value for “Preoperative”< “First diagnosed”.

Longitudinal assessment of tumor markers (CEA, CA19-9, and AFP) before and after NAT revealed no significant differences between the NACI and NAC groups, either in baseline levels or treatment-induced changes (all P > 0.05) (**Table 4**).

**Table 4:** Comparison of tumor markers and adverse events during neoadjuvant therapy.

Baseline variable	NAC group	NACI group	χ²/Z	P value
	(n=207) %	(n=168) %		
CEA①			Z=-0.071	0.943
Median (IQR), ng/mL	2.6(1.7,6.1)	2.65(1.6,6.1)		
CA19-9①			Z=-0.567	0.571
Median (IQR), U/mL	9.0(5.3,20.0)	12.85(6.3,33.6)		
AFP①			Z=-1.437	0.151
Median (IQR), ng/mL	3.0(2.2,4.0)	2.6(1.9,4.0)		
CEA②			Z=-1.299	0.194
Median (IQR), ng/mL	2.8(1.9,4.1)	2.3(1.7,4.0)		
CA19-9②			Z=-0.352	0.725
Median (IQR), U/mL	9.8(5.3,16.0)	9.9(5.15,16.8)		
AFP②			Z=-1.218	0.223
Median (IQR), ng/mL	3.0(2.2,4.2)	2.9(2.0,4.3)		
▵CEA			Z=-0.121	0.904
Median (IQR), ng/mL	0.1(-0.5,1.6)	0.05(-0.6,1.8)		
▵CA19-9			Z=-0.650	0.515
Median (IQR), U/mL	0.5(-1.7,5.1)	1.2(-4.2,15.9)		
▵AFP			Z=-0.101	0.920
Median (IQR), ng/mL	-0.06(-0.6,0.5)	-0.1(-1.0,0.7)		
Leukocyte decreased			χ²=2.932	0.087
Grade 0,1	191(92.2%)	146(86.9%)		
Grade 2,3,4	16(7.7%)	22(13.1%)		
Neutrophils decrease			χ²=0.781	0.377
Grade 0,1	177(85.5%)	138(82.1%)		
Grade 2,3,4	30(14.5%)	30(17.9%)		
Lymphocyte decrease			χ²=0.058	0.810
Grade 0,1	184(88.9%)	148(88.1%)		
Grade 2,3,4	23(11.1%)	20(11.9%)		
Hemoglobin decrease			χ²=0.889	0.345
Grade 0,1	145(70.0%)	110(65.4%)		
Grade 2,3,4	62(30.0%)	58(34.6%)		
Platelets decrease			χ²=0.000	1
Grade 0,1	203(98.0%)	165(98.2%)		
Grade 2,3,4	4(1.9%)	3(1.8%%)		
ALT/AST increase			χ²=1.002	0.317
Grade 1	16(7.7%)	18(10.7%)		
▵Leukocyte	26(12.6%)	33(19.6%)	χ²=3.509	0.061
▵Neutrophil	43(20.8%)	34(20.2%)	χ²=0.016	0.899
▵Lymphocyte	39(18.8%)	34(20.2%)	χ²=0.116	0.734
▵Hemoglobin	53(25.6%)	55(32.7%)	χ²=2.302	0.129
▵Platelets	6(2.9%)	10(6.0%)	χ²=2.117	0.146
▵ALT/AST	12(5.8%)	17(10.1%)	χ²=2.428	0.110

①Tumor markers at first diagnosis.

②Preoperative tumor markers.

CEA, Carcinoembryonic antigen; CA199, Carbohydrate antigen199; AFP, Alpha-fetoprotein; ALT, alanine aminotransferase; AST, Aspartate aminotransferase; NAC, neoadjuvant chemotherapy; NACI, Neoadjuvant chemotherapy plus immunotherapy.

▵CEA, ▵CA19-9, and ▵AFP represent the values that increase or decrease after neoadjuvant therapy.

▵Leu, ▵Neu, ▵Neu, ▵Hb, and ▵PLT represent the number of cases with increased decline and grade after neoadjuvant therapy, and ▵ALT/AST represents the number of cases with increased grade after neoadjuvant therapy.

Z is a negative value for “NACI group”< “NAC group”.

Hematologic toxicities predominated among treatment-related adverse events, with leukopenia, neutropenia, lymphopenia, anemia, thrombocytopenia, and transaminase elevation (ALT/AST) being most frequently observed. The overall incidence and severity of adverse events were comparable between two groups (all P > 0.05) (**Table 4**). Notably, no 30-day postoperative mortality or unplanned reoperations were recorded in either treatment arm.

### 3.4 Pathological response and correlation analysis

Histopathological evaluation revealed superior tumor regression in the NACI cohort, with significantly higher rates of TRG (1a+1b) responses compared to the NAC group (31.0% vs. 18.8%, P = 0.007). This difference was particularly pronounced in the TRG 1a subgroup (20.2% vs. 12.6%, P = 0.04). Furthermore, the NACI regimen was associated with enhanced pathological downstaging, evidenced by increased frequencies of ypT0 (22.0% vs. 13.0%, P = 0.022) and ypN0 (67.3% vs. 53.6%, P = 0.007) statuses. Notably, the median total lymph node yield was substantially greater in the NACI arm (24 [IQR 18–28] vs. 19 [15–24], P < 0.001), accompanied by a reduced burden of metastatic lymph nodes (0 [0–1.75] vs. 0 [0–5], P = 0.001). However, no intergroup differences were observed in tumor differentiation grade, lymphovascular invasion, or perineural invasion (all P > 0.05) (**Table 5**). The NACI group achieved a significantly higher R0 resection rate compared to the NAC group (91.3% vs. 84.1%, P = 0.028), underscoring the potential superiority of this regimen in ensuring complete tumor removal (**Table 6**). Univariate analysis identified several clinical and pathological factors significantly associated with TRG (1a+1b) response, including age (P < 0.05), pretreatment lymph node positivity (P < 0.05), PD-1 inhibitor administration (P < 0.05), tumor differentiation grade (P < 0.05), and lymph node metastasis (P < 0.05). Multivariate logistic regression confirmed that poor tumor differentiation (RR = 3.397, 95% CI: 1.361–8.475, P = 0.009) and higher metastatic lymph node burden (RR = 1.802, 95% CI: 1.074–3.024, P = 0.026) were independent risk factors (**Table 7**).

**Table 5:** Pathological response and tumor characteristics.

Baseline variable	NAC group	NACI group	χ^2^/Z	P value
	(n=207) %	(n=168) %		
TRG			Z=-3.268	0.001
TRG 1a/pCR	26(12.6%)	34(20.2%)	χ²=4.067	0.04
TRG 1b	13(6.2%)	18(10.7%)		
TRG 2	26(12.6%))	37(22.0%)		
TRG 3	142(68.6%)	79(47.1%)		
MPR			χ²=7.402	0.007
Yes	39(18.8%)	52(31.0%)		
No	168(81.2%)	116(69.0%)		
Differentiation			Z=-0.578	0.563
Well	12(5.8%)	7(4.2%)		
Middle	59(28.5%)	40(23.8%)		
Poorly differentiated and undifferentiated	109(52.7%)	82(48.8%)		
ypT stage			Z=-3.032	0.002
ypT0	27(13.0%)	37(22.0%)	χ²=5.284	0.022
ypT1	12(5.8%)	20(11.9%)		
ypT2	28(13.5%)	37(22.0%)		
ypT3	69(32.9%)	41(24.4%)		
ypT4a	52(25.1%)	22(13.1%)		
ypT4b	20(9.7%)	11(6.6%)		
ypN stage			Z=-1.898	0.058
ypN0	111(53.6%)	113(67.3%)	χ²=7.172	0.007
ypN1	16(7.7%)	16(9.5%)		
ypN2	46(22,2%)	26(15.4%)		
ypN3a	26(12.6%)	10(6.0%)		
ypN3b	8(3.9%)	3(1.8%)		
Harvested lymph nodes			Z=-4.402	<0.001
Median (IQR)	19(15,24)	24(18,28)		
Positive lymph nodes			Z=-3.249	0.001
Median (IQR)	0(0,5)	0(0,1.75)		
Vessel invasion			χ²=2.791	0.095
Yes	24(11.6%)	11(6.6%)		
No	183(88.4%)	157(93.4%)		
Nerve invasion			χ²=0.320	0.572
Yes	22(10.6%)	21(12.5%)		
No	185(89.4%)	147(87.5%)		

NAC, neoadjuvant chemotherapy; NACI, Neoadjuvant chemotherapy plus immunotherapy.

**Table 6:** R0 resection rates.

	NAC group	NACI group	χ^2^/Z	p value
	(n=246) %	(n=184) %		
R0 resection	207(84.1%)	168(91.3%)	χ²=4.835	0.028

NAC, neoadjuvant chemotherapy; NACI, Neoadjuvant chemotherapy plus immunotherapy.

**Table 7:** Univariate and multivariate analysis of factors associated with tumor regression grade (TRG).

Baseline variable	TRG(1a+1b)(n=91)	TRG(2 + 3)(n=284)	P value	RR	95%CI	P value
Gender			0.383			
Male	59	198				
Female	32	86				
Age			0.027	Ref 0.974	0.939-1.01	0.156
BMI			0.976			
cN			0.011	Ref 2.155	0.82-5.662	0.119
cN+	66	240				
cN0	25	44				
Combination with PD-1 inhibitors			0.007	Ref 0.667	0.277-1.608	0.367
Yes	52	116				
No	39	168				
Neoadjuvant cycles			0.539			
>3	12	142				
≤3	79	142				
Duration of operation			0.794			
Estimated blood loss			0.947			
Complications			0.181			
Yes	20	45				
No	71	239				
Differentiation			0.003	Ref 3.397	1.361-8.475	0.009
Well and Middle	17	101				
Poorly differentiated and undifferentiated	8	183				
Harvested lymph nodes			0.342			
Positive lymph nodes			<0.001	Ref 1.802	1.074-3.024	0.026

### 3.5 Horizontal comparison of PD-1 inhibitors

Within the NACI cohort, no statistically significant differences were observed among patients treated with camrelizumab, sintilimab, nivolumab, or tislelizumab in terms of TRG grading, tumor differentiation, ypT0, ypN0, total lymph node dissection, lymph node metastasis, vascular invasion, or perineural invasion (all P > 0.05) (**Table 8**).

**Table 8:** Comparison of pathological outcomes among different PD-1 inhibitors in the NACI group.

	Sintilimab	Camrelizumab	Nivolumab	Tislelizumab	χ²/H	P value
	(n=24) %	(n=68) %	(n=14) %	(n=62) %		
TRG					χ²=0.718	0.874
TRG 1a+1b	7(29.2%)	19(27.9%)	5(35.7%)	21(33.9%)		
TRG 2 + 3	17(70.8%)	49(72.1%)	9(64.3%)	41(62.1%)		
Differentiation					H=1.973	0.583
Well	1(4.2%)	3(4.4%)	0(0.0%)	3(4.8%)		
Middle	4(16.7%)	16(23.5%)	4(28.6%)	16(25.8%)		
Poorly differentiated and undifferentiated	16(66.6%)	34(50.0%)	6(42.8)	26(41.9%)		
ypT stage					χ²=0.948	0.814
ypT0	3(12.5%)	15(22.0%)	3(21.4%)	16(25.8%)		
ypT+						
ypN stage					χ²=5.695	0.127
ypN0	11(45.8%)	47(69.1%)	10(71.4%)	45(73.6%)		
ypN+						
Harvested lymph nodes					H=3.155	0.368
Median (IQR)	25(19,36)	22(16,27)	22(16.75,30.5)	24(18,29)		
Positive lymph nodes					H=0.291	0.962
Median (IQR)	0(0,3)	0(0,1)	0(0,2,25)	0(0,0)		
Vessel invasion					Fisher	0.344
Yes	2(8.3%)	2(2.9%)	1(7.1%)	6(9.7%)		
No	22(91.7%)	66(97.1%)	13(92.9%)	56(90.3%)		
Nerve invasion					χ²=2.908	0.465
Yes	2(8.3%)	6(8.8%)	3(21.4%)	10(16.1%)		
No	22(91.7%)	62(91.2%)	11(78.6%)	52(83.9%)		
pCR	3(12.5%)	14(20.6%)	3(21.4%)	14(22.6%)	χ²=1.631	0.684

### 3.6 Long-term survival and COX regression analysis

**Figure 2:**
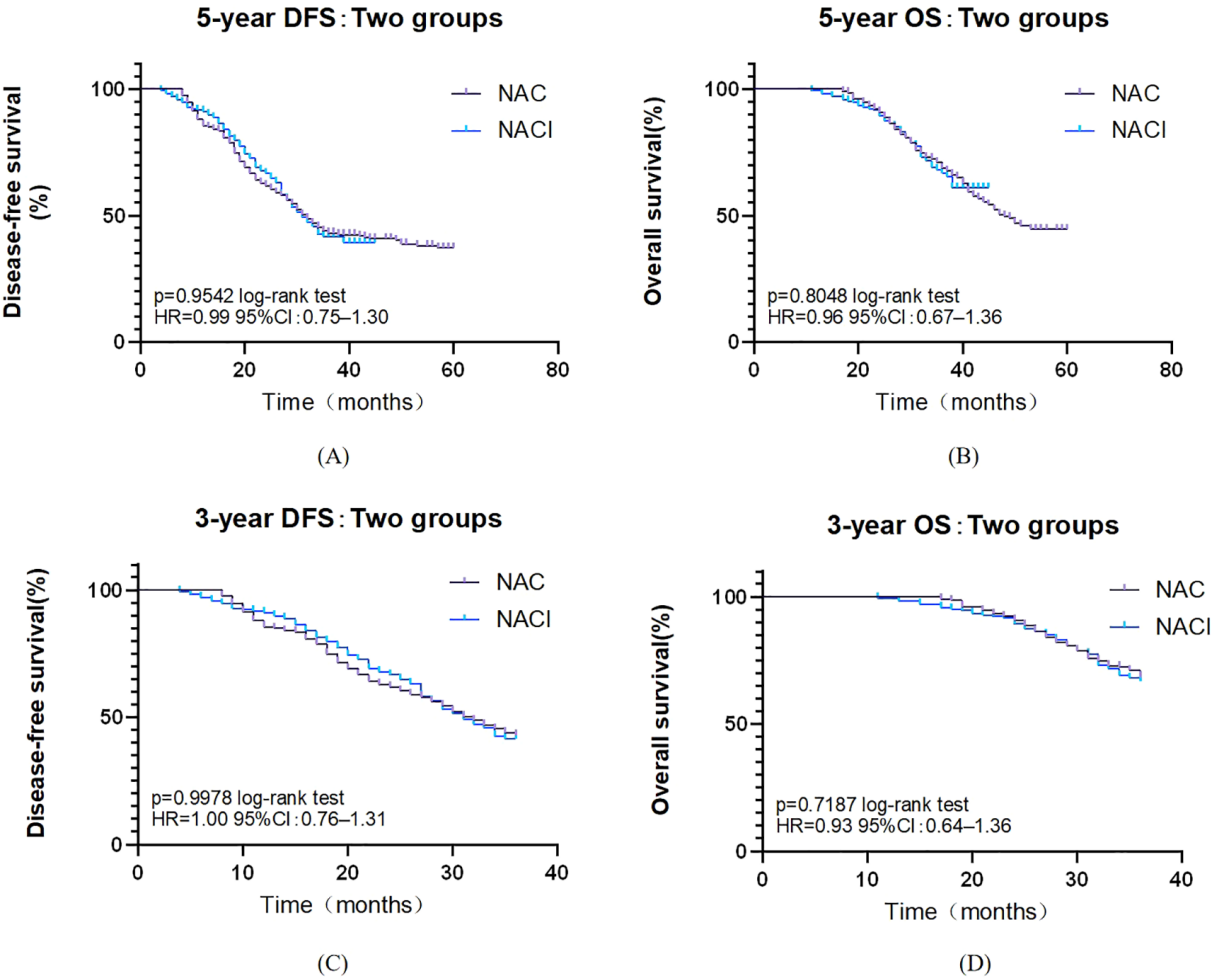
**(A)** Survival curve of overall 5-year DFS. **(B)** Survival curve of overall 5-year OS. **(C)** Survival curve of overall 3-year DFS. **(D)** Survival curve of overall 3-year OS.

All 375 patients completed follow-up for survival analysis. The median follow-up duration was 36 months (intergroup difference: 33 months in the NACI group vs. 42 months in the NAC group; P < 0.001). Due to the significant difference in follow-up time between groups, it was decided to give priority to whether there was a difference in 5-year survival between the NAC group and the NACI group. The median 5-year DFS was comparable between the NAC group (32.0 months, 95% CI: 28.0–35.9) and the NACI group (31.0 months, 95% CI: 27.7–34.3), with no statistically significant difference observed (HR = 0.99, 95% CI: 0.75–1.30, P = 0.954). Similarly, the median 5-year OS in the NAC group was 48.0 months (95% CI: 42.1–53.9), while it was not reached in the NACI group, and no significant difference was detected (HR = 0.96, 95% CI: 0.67–1.36, P = 0.805). Because the first patient receiving NACI in our hospital had surgery time in July 2021 and the follow-up cut-off time was April 2025, the longest follow-up in the NACI group was only 45 months, and the median follow-up time in the NACI group was 31 months, so we further evaluated the 3-year survival rate. No significant difference was found between 3-year DFS (P = 0.968) or 3-year OS (P = 0.719). (**Figure 2**). Cox survival analysis in this study demonstrated that ypN+ (HR = 2.179, 95%CI: 1.615-2.94, P<0.001), pCR (HR = 0.218, 95%CI: 0.097-0.493, P<0.001), number of positive lymph nodes (HR = 1.061, 95%CI: 1.037-1.086, P<0.001), and vascular invasion (HR = 2.124, 95%CI: 1.416-3.186, P<0.001) were significantly associated with OS, while PD-1 inhibitor combination therapy (HR = 0.621, 95%CI: 0.471-0.819, P<0.001), ypN+ (HR = 1.309, 95%CI: 1.015-1.688, P = 0.038), and number of positive lymph nodes (HR = 1.026, 95%CI: 1.003-1.049, P = 0.029) showed significant correlations with DFS; multivariate analysis confirmed ypN+ (HR = 1.548, 95%CI: 1.057-2.268, P = 0.025) as a poor prognostic factor for OS, and pCR (HR = 0.304, 95%CI:0.132-0.702, P = 0.005) was a protective factor for OS, whereas PD-1 inhibitor combination (HR = 0.573, 95%CI: 0.432-0.761, P<0.001) remained significantly associated with improved DFS (**Tables 9**, **10**).

**Table 9:** Cox regression analysis for overall survival.

Baseline variable	Univariate analyze		Multivariate analyze	
	HR(95%CI)	P value	HR(95%CI)	P value
Gender	0.816(0.582-1.143)	0.237		
Age	0.999(0.987-1.012)	0.894		
BMI	0.998(0.957-1.041)	0.933		
Combination with PD-1 inhibitors	1.045(0.735-1.486)	0.806		
Neoadjuvant cycle >3	0.931(0.618-1.404)	0.733		
Duration of operation	0.999(0.997-1.001)	0.464		
Estimated blood loss	1(1-1.001)	0.269		
Complications	1.183(0.797-1.756)	0.405		
Poorly differentiated and undifferentiated	1.219(0.886-1.677)	0.223		
ypN+	2.179(1.615-2.94)	<0.001	1.548(1.057-2.268)	0.025
pCR	0.218(0.097-0.493)	<0.001	0.304(0.132-0.702)	0.005
Harvested lymph nodes	1.005(0.989-1.022)	0.524		
Positive lymph nodes	1.061(1.037-1.086)	<0.001	1.018(0.983-1.055)	0.316
Vascular invasion	2.124(1.416-3.186)	<0.001	1.465(0.939-2.286)	0.092
Nerve invasion	1.537(0.996-2.371)	0.052		

**Table 10:** Cox regression analysis for disease-free survival.

Baseline variable	Univariate analyze		Multivariate analyze	
	HR(95%CI)	P value	HR(95%CI)	P value
Gender	1.147(0.865-1.521	0.341		
Age	0.988(0.975-1)	0.059		
BMI	0.999(0.965-1.035)	0.957		
Combination with PD-1 inhibitors	0.621(0.471-0.819)	<0.001	0.573(0.432-0.761)	<0.001
Neoadjuvant cycle >3	0.874(0.610-1.252)	0.462		
Duration of operation	1.002(0.999-1.004)	0.162		
Estimated blood loss	1(0.999-1.001)	0.826		
Complications	1.110(0.794-1.551)	0.541		
Poorly differentiated and undifferentiated	1.100(0.834-1.451)	0.499		
ypN+	1.309(1.015-1.688)	0.038	1.244(0.905-1.709)	0.179
pCR	0.770(0.448-1.324)	0.345		
Harvested lymph nodes	1.002(0.988-1.017)	0.753		
Positive lymph nodes	1.026(1.003-1.049)	0.029	1.022(0.992-1.053)	0.155
Vascular invasion	1.186(0.824-1.707)	0.358		
Nerve invasion	1.361(0.943-1.965)	0.099		

## 4 Discussion

In this study, we assessed the safety, pathological outcomes, and long-term prognosis of laparoscopic gastrectomy following NAC or NACI. Safety analyses revealed comparable rates of treatment-related adverse events between the NACI and NAC groups, demonstrating that PD-1 blockade does not amplify toxicity during NAT. Moreover, operative metrics—including duration, blood loss, postoperative complications, and hospitalization length—showed no significant intergroup differences. These results suggest that immune checkpoint inhibition does not compromise the feasibility or safety of minimally invasive gastrectomy, aligning with prior reports (26–28). Notably, comparable postoperative recovery trajectories (29, 30) further underscore the short-term safety of NACI, reinforcing its potential for integration into multimodal treatment strategies.

In this study, AFP testing was incorporated to screen for hepatoid differentiation or occult liver metastases. While AFP exhibits limited sensitivity in GC, aberrant elevation may indicate aggressive biological behavior, such as hepatoid adenocarcinoma, which is typically associated with poor prognosis (31). However, no significant AFP fluctuations were observed in this cohort, potentially due to the low prevalence of AFP-positive cases or the limited role of AFP secretion in non-hepatoid GC subtypes. Notably, emerging evidence highlights the prognostic value of preoperative CEA and CA19-9 levels in GC. Elevated CEA/CA19-9 has been linked to unfavorable outcomes, whereas a marked decline following NAT correlates with improved survival (32, 33). In our study, the significant reduction in CEA levels (P < 0.05) during NAT aligned with a higher pathological response rate in the NACI group (34), reinforcing its utility as a potential predictor of treatment efficacy. Moving forward, serial monitoring of these biomarkers could help identify early responders or high-risk patients prone to relapse. The clinical relevance of AFP in GC warrants further validation, particularly within AFP-positive subgroups.

Our study demonstrates that NACI for LAGC is not only safe but also confers significant short-term therapeutic advantages. The primary endpoint, pCR rate, and secondary endpoint, MPR rate, were both markedly enhanced in the NACI cohort compared to chemotherapy alone. Previous studies have demonstrated that pathological response is a reliable marker for short-term efficacy following neoadjuvant therapy, with pCR achievement strongly correlating with improved long-term survival outcomes (35). Historically, the pCR rate among LAGC patients receiving NACT alone averages around 6.7%, whereas those receiving combined NACT and immunotherapy have reported pCR rates ranging from 19.4% to 33.6% (23, 30, 36, 37). In our study, the pCR rate reached 20.2%, significantly higher than in NAC alone cohorts and consistent with prior research. This supports the hypothesis that immunotherapy enhances antitumor activity by activating T-cell-mediated immune responses, thereby augmenting the cytotoxic effects of chemotherapy (12, 13). Although no significant difference in clinical T/N (cT/cN) stage was observed between the two groups, the NACI group exhibited a marginally higher proportion of cT4 patients (75.0% vs. 73.4%). Notably, prior studies have reported a significant decline in R0 resection rates among T4-stage gastric cancer patients (38). However, the potential sensitivity of certain tumor subsets (e.g., MSI-H or PD-L1-high subgroups) to immunotherapy (14, 39) may counteract the adverse effects of advanced staging on R0 resection. Due to incomplete genetic profiling in most included patients, the influence of these subgroups could not be definitively assessed. Thus, these findings warrant further validation in cohorts with comprehensive molecular characterization. The NACI group demonstrated a higher proportion of upper gastric cancer cases compared to the control group (23.2% vs. 21.2%). Intriguingly, prior studies suggest that upper gastric cancer may exhibit enhanced pathological responsiveness to neoadjuvant chemotherapy (40), potentially influencing treatment outcomes in this cohort. Previous studies have demonstrated that PD-1 inhibitors can potentiate the cytotoxic effects of chemotherapy against primary tumors and micrometastases by augmenting T cell-mediated immune responses. This synergistic action not only reduces tumor invasiveness but also enhances the likelihood of achieving R0 resection (41, 42). The R0 resection rate was higher in the NACI group (91.3% vs. 84.1%), aligning with findings from Lin et al. (26, 30), although Cui et al (23, 43) have argued that PD-1 inhibitors do not necessarily increase R0 resection rates. Patients with ypN+ status exhibited poorer overall survival, whereas achieving ypN0 status remains a critical indicator of successful preoperative treatment in gastric cancer, independent of cN status (44). Our findings demonstrate that NACI induced significantly greater tumor downstaging compared to chemotherapy alone, with markedly higher rates of ypT0 (complete pathological regression) and ypN0 (node-negative status; both p<0.05). This enhanced pathological response suggests improved oncological outcomes and supports the potential of NACI to convert initially unresectable or borderline-resectable tumors to operable status. These results align with subgroup analyses from the KEYNOTE-585 phase III trial (45).

This study demonstrated that the NACI group exhibited a significantly higher number of dissected lymph nodes (P<0.001) alongside a lower proportion of positive lymph nodes (P = 0.001) compared to the NAC group, a finding that contrasts with prior reports (27, 28, 46). First, the NACI group exhibited a slightly higher rate of total gastrectomy compared to the NAC group (60.7% vs. 55.6%). Given that total gastrectomy typically necessitates more extensive lymphadenectomy (47), this could partially account for the increased lymph node retrieval. Additionally, the NACI group had a marginally higher proportion of upper gastric cancers (23.2% vs. 21.2%). Since proximal tumors demonstrate greater propensity for lymph node metastasis and often require extended D2+ dissection (48, 49), this may further contribute to the elevated lymph node count. However, no significant intergroup differences were observed in surgical approach (P = 0.526) or tumor location (P = 0.246), necessitating further validation through prospective, multicenter studies. Second, in retrospective analyses, lymph node detection may be influenced by variations in surgical expertise and pathological processing. Since NACI has been carried out in our hospital in recent years, it was carried out later than NAC, surgeons operating on NACI patients may possess higher proficiency, potentially leading to more comprehensive lymph node harvesting. Although standardized pathological protocols were employed, interobserver variability cannot be entirely excluded. Future prospective studies should harmonize surgical skill levels and further refine lymph node processing and pathological evaluation to mitigate bias. Finally, if the NACI cohort includes a higher prevalence of immunotherapy-sensitive subtypes (e.g., MSI-H or PD-L1-high tumors), immune activation within lymph nodes might enhance the detection of micrometastases (14, 39, 41), indirectly increasing nodal yield. Molecular subtyping and stratified analyses in future studies are warranted to clarify this potential confounding effect.

In both univariate and multivariate analyses of TRG 1a/1b, the use of PD-1 inhibitors was not identified as an independent protective factor. This finding contrasts with the results reported by Bao et al. (50), highlighting a need for further investigation to resolve this discrepancy. Moreover, intra-group comparisons within the NACI cohort demonstrated no statistically significant differences in pCR rates, MPR rates, tumor downstaging (ypT0/ypN0 rates), or lymph node metastasis counts among the four treatment groups (sintilimab, camrelizumab, nivolumab, and tislelizumab). Given these results, multicenter, large-scale studies are warranted to validate these observations and clarify potential inter-agent variability.

With a median follow-up of 36 months, 33 months in the NACI group, and 42 months in the NAC group, and there was a significant difference in median follow-up between the two groups (P<0.001), which was due to the fact that the first patient receiving NACI in our hospital had a surgical time of July 2021, a follow-up cut-off time of April 2025, and a maximum follow-up time of only 45 months in the NACI group, so we further evaluated the 3-year survival rate. Although the NACI group did not demonstrate a significant survival advantage in this study, these findings align with the preliminary results of KEYNOTE-585 (45). Similarly, the MATTERHORN trial (51) reported a marked improvement in pCR rates (19.2% vs. 7.2%) without a statistically significant difference in 2-year event-free survival. This suggests that extended follow-up or biomarker stratification (e.g., PD-L1-positive populations) may be necessary to elucidate potential survival benefits. Multivariate Cox regression analysis demonstrated that incorporating PD-1 inhibitors into NAT significantly improved DFS (HR = 0.573, P=0.005). These findings align with emerging evidence supporting the perioperative use of ICIs, particularly as demonstrated in the landmark MATTERHORN trial (51). Our COX multivariate analysis demonstrated a robust correlation between pCR and OS (HR = 0.304, P<0.001). These findings mirror the therapeutic benefits observed in the MATTERHORN trial, where durvalumab treatment yielded both superior pCR rates (19.2% vs 7.2%) and enhanced event-free survival in the perioperative setting. In the present study, while PD-1 inhibition significantly enhanced DFS (HR = 0.573), the absence of OS benefit may reflect insufficient follow-up duration or crossover effects from subsequent therapies—a phenomenon also observed in CheckMate 577 (52). Further investigation with longer follow-up and biomarker-driven patient selection is warranted to clarify the survival impact of immune-based neoadjuvant strategies.

This study has certain limitations and deficiencies. Firstly, as a single-center retrospective analysis with a modest sample size, the findings may be influenced by selection bias and unmeasured confounding factors, potentially leading to overestimated hazard ratios. The study cohort was restricted to patients who underwent R0 resection for gastric cancer and completed adjuvant chemotherapy, which may limit the generalizability of results to broader LAGC populations. Secondly, the follow-up time between the groups was unbalanced (p<0.05), because the first patient receiving NACI in our hospital had a surgical time of July 2021 and a follow-up deadline of April 2025, so the longest follow-up in the NACI group was only 45 months, and the median follow-up time was only 31 months. Consequently, long-term survival outcomes require further validation. Third, the homogeneity of chemotherapy regimens precluded evaluation of protocol-specific efficacy differences. Finally, critical questions—including the optimal number of immunotherapy cycles and ideal surgical timing to maximize immune activation—remain unresolved and warrant investigation in prospective trials. Future studies should prioritize multicenter randomized controlled designs incorporating dynamic biomarkers (e.g., CD8+ T cell infiltration) to assess tumor immune microenvironment evolution and guide personalized therapy.

## 5 Conclusions

Our findings suggest that laparoscopic surgery following NACI may offer a safe and effective treatment strategy for LAGC patients. The NACI regimen demonstrated significant improvements in pCR and R0 resection rates while maintaining favorable surgical safety profiles and postoperative recovery timelines. However, multicenter phase III randomized controlled trials with larger cohorts are warranted to validate these findings and determine whether NACI confers durable clinical benefits in terms of DFS and OS.

## Data Availability

The original contributions presented in the study are included in the article/supplementary material. Further inquiries can be directed to the corresponding author.
